# Draft Genome Sequence of Escherichia marmotae E690, Isolated from Beef Cattle

**DOI:** 10.1128/MRA.00739-20

**Published:** 2020-08-06

**Authors:** Medelin Ocejo, Maitane Tello, Beatriz Oporto, José Luis Lavín, Ana Hurtado

**Affiliations:** a Animal Health Department, NEIKER–Basque Institute for Agricultural Research and Development, Basque Research and Technology Alliance (BRTA), Derio, Bizkaia, Spain; b Applied Mathematics Department, Bioinformatics Unit, NEIKER–Basque Institute for Agricultural Research and Development, Basque Research and Technology Alliance (BRTA), Derio, Bizkaia, Spain; University of Maryland School of Medicine

## Abstract

We report here the draft genome sequence of an extended-spectrum β-lactamase (ESBL)-producing *Escherichia* species isolated from rectal feces collected from beef cattle in northern Spain. Analysis of the draft genome identified the strain as a member of the newly described species Escherichia marmotae.

## ANNOUNCEMENT

The genus *Escherichia* (family *Enterobacteriaceae*) includes four species, Escherichia coli (1), Escherichia fergusonii (2), Escherichia albertii (3), and the recently described Escherichia marmotae (4, 5), along with several cryptic clades (6).

We report here the draft genome sequence of *E. marmotae* strain E690, recovered in 2016 from rectal feces collected from beef cattle in the Basque Country (northern Spain) as part of a cross-sectional survey conducted to estimate the herd prevalence of extended-spectrum β-lactamase/AmpC-producing E. coli in ruminants (7). Feces (25 g) diluted 1:10 in modified tryptic soy broth (bioMérieux) supplemented with novobiocin (Biolife) was incubated at 41 ± 1°C (6 to 7 h), preenriched in MacConkey broth with 1 mg/liter cefotaxime (37 ± 1°C, 24 h), and subcultured onto MacConkey agar with cefotaxime (1 mg/liter).

DNA extracted from pure culture (Wizard genomic DNA purification kit, Promega) was submitted to Eurofins Genomics, where libraries were prepared using the NEBNext Ultra II FS DNA library prep kit (Illumina). The genome was sequenced using an Illumina NovaSeq 6000 instrument (150-bp paired-end reads), resulting in 23,934,128 reads (718× coverage). Quality control was assessed using FastQC v.0.11.9 (8) and then analyzed via TORMES v.1.0 (9). Briefly, the reads were quality filtered using Trimmomatic (10) and PRINTSEQ (11) and *de novo* assembled using SPAdes (12). The quality of the assemblies was assessed with QUAST (13), discarding contigs below 200 bp with PRINSEQ. BLASTn v.2.9.1+ (14) and ABRicate were used to screen for acquired antimicrobial resistance genes in ResFinder (15), chromosomal point mutations associated with antimicrobial resistance in PointFinder (16), and virulence genes in the Virulence Factors Database (VFDB) (17). Plasmid replicons were identified using PlasmidFinder (18). PlasFlow (19) predicted plasmid- and chromosome-derived contigs. *In silico* FimH typing was achieved using FimTyper (20). Multilocus sequence types (MLSTs) (Achtman scheme) were queried against the E. coli MLST database PubMLST (21) using mlst, and the core-genome MLST (cgST) was assigned using cgMLSTFinder, following the EnteroBase E. coli cgMLST scheme (22). Gene identification and annotation were retrieved from the NCBI Prokaryotic Genome Annotation Pipeline (PGAP) (23). See Table 1 for versions/commands used for specific tools.

**TABLE 1:** Bioinformatics tools used in the study along with the versions, scripts, and references

Tool	Version	Script(s) run on command line	Reference or source
Trimmomatic	0.38	trimmomatic PE -phred33 Raw_reads/Sample_R1.fastq.gz Raw_reads/Sample_R2.fastq.gz /cleaned_reads/Sample_noadapt.R1.fastq.gz /dev/null cleaned_reads/Sample_noadapt.R2.fastq.gz /dev/null ILLUMINACLIP: adapters.fa:1:30:1	10
PRINSEQ	0.20.4	raw data quality filtering: perl prinseq-lite.pl -verbose -fastq cleaned_reads/Sample_noadapt.R1.fastq -fastq2 cleaned_reads/Sample_noadapt.R2.fastq -out_good cleaned_reads/Sample_ok -out_format 3 -out_bad null -min_len 125 -min_qual_mean 25 -trim_qual_right 25 -trim_qual_window 15 -trim_qual_type mean	11
		contigs filtering: perl prinseq-lite.pl -fasta assembly/Sample_assembly/contigs.fasta -min_len 200 -out_good genomes/Sample -out_bad null	
SPAdes	3.13.0	python spades.py --careful -1 cleaned_reads/Sample_ok_1.fastq.gz -2 cleaned_reads/Sample_ok_2.fastq.gz -o assembly/Sample_assembly -t 8	12
QUAST	5.0.2	quast genomes/Sample.fasta -o /genome_stats/Sample_genome_stats -t 16 --min-contig 200 --no-icarus --silent --no-sv	13
ABRicate	0.8.10	abricate genomes/Sample.fasta --db db* –nopath (in the curation of the results table, all hits with coverage below 60% and identity below 90% were removed) *db = Resfinder, PlasmidFinder, VFDB	https://github.com/tseemann/abricate
PointFinder	3.1.0	python PointFinder.py -i genomes/Sample.fasta -p pointfinder_db -m blastn -m_p blastn -s ecoli -o point_mutations/Sample	16
PlasFlow	1.1	PlasFlow.py --input genomes/Sample.fasta --output PlasFlow/ Sample.predictions --threshold 0.7	19
FimTyper	1.1	perl fimtyper.pl -d fimtyper_db -b ncbi-blast-2.9.0+/ -i genomes/Sample.fasta -o /fimH_typing/Sample -k 95.00 -l 0.80	20
mlst	2.16.1	mlst genomes/Sample.fasta --nopath --quiet > mlst/mlst.tab	https://github.com/tseemann/mlst
cgMLSTFinder	1.1	docker run --rm -it -v $(workdir):/workdir cgmlstfinder -o output -s ecoli -db cgmlstfinder_db -t temp cleaned_reads/Sample.fastq.gz	https://bitbucket.org/genomicepidemiology/cgmlstfinder/src/master/
PGAP	4.11 2020-03-30.build4489	./pgap.py -r -o Sample_results genomes/Sample/input.yaml	23

The chromosome sequence length was 4,303,797 bp (63 contigs; *N*_50_, 175,886 bases), with a 50.4% G+C content. The chromosome contains 4,094 genes (3,951 protein-coding, 74 pseudogenes, and 69 RNA genes). Moreover, two plasmid incompatibility group-determining sequences were identified, IncFI and IncI1. Pairwise comparisons of the E690 genome versus closely related strain genomes performed at the Type (Strain) Genome Server (TYGS) (24) identified the genome of *E. marmotae* HT073016^T^ as the closest match. Intergenomic comparison of E690 and *E. marmotae* HT073016^T^ by digital DNA-DNA hybridization (d_4_ = 94.6 [93.0 to 95.9]) and G+C content (0.18 difference) identified strain E690 as *E. marmotae*.

MICs were determined by broth microdilution following the recommendations in Commission Decision 2013/652/EU using two Sensititre MIC susceptibility plates (EUVSEC1 and EUVSEC2, Thermo Fisher Scientific). *E. marmotae* E690 exhibits microbiological resistance to tetracycline (MIC > 64 mg/liter) and the β-lactams ampicillin (MIC > 64 mg/liter), cefotaxime (MIC = 8 mg/liter), ceftazidime (MIC = 16 mg/liter), and cefepime (MIC = 1 mg/liter) and carries the *tet*(A) and *bla*_SHV-12_ genes in an IncI1 plasmid. It carries mutations in the topoisomerase genes, *parC* (p.S57T) and *parE* (p.I355T) but is susceptible to nalidixic acid (MIC < 4 mg/liter) and ciprofloxacin (MIC = 0.03 mg/liter). It belongs to MLST ST-6495 and cgST-141216 and carries a *fim*H160 allele. *E. marmotae* E690 harbors virulence factors related to extraintestinal pathogenic E. coli (ExPEC), like F1 fimbriae, the K1 capsule, and the protein OmpA (chromosomally encoded), and to animal enterotoxigenic E. coli (ETEC), like F4 fimbriae and a heat-stable enterotoxin EAST1 (plasmid encoded).

### Data availability.

The genome sequence was deposited under GenBank accession number JABXGM000000000, BioProject accession number PRJNA632731, and BioSample accession number SAMN14918579.
